# Discriminating from species of *Curcumae* Radix (*Yujin*) by a UHPLC/Q-TOFMS-based metabolomics approach

**DOI:** 10.1186/s13020-016-0095-8

**Published:** 2016-04-29

**Authors:** Fang Liu, Xu Bai, Feng-Qing Yang, Xiao-Jing Zhang, Yuanjia Hu, Peng Li, Jian-Bo Wan

**Affiliations:** State Key Laboratory of Quality Research in Chinese Medicine, Institute of Chinese Medical Sciences, University of Macau, Macao, People’s Republic of China; Waters Technologies (Shanghai) Ltd., Shanghai, People’s Republic of China; School of Chemistry and Chemical Engineering, Chongqing University, Chongqing, 400030 People’s Republic of China

## Abstract

**Background:**

Chinese medicinal herbs may use more than one species of *Curcumae* Radix (*Yujin*) is the tuberous roots of *Curcumae wenyujin*, *C. kwangsiensis*, *C. phaeocaulis* and *C. longa*. This study aimed to characterize the chemical profiles of these different species of *Curcumae* Radix, and develop a method for rapid discrimination of these species by ultra-high performance liquid chromatography–quadrupole time-of-flight mass spectrometry (UHPLC/Q-TOFMS) combined with multivariate statistical analysis.

**Methods:**

The metabolomes of 33 different batches of *Curcumae* Radix derived from four *Curcumae* species were profiled by UHPLC/Q-TOFMS. The resulting sample codes, *t*_R_–*m/z* pairs and ion intensities were processed by unsupervised principal component analysis (PCA) and supervised orthogonal partial least squared discriminant analysis (OPLS-DA) to characterize the chemical composition of *Curcumae* Radix across the four different species.

**Results:**

Obvious differences were observed in the chemical compositions of the *Curcumae* Radix samples derived from the four different species according to PCA and OPLS-DA. These results suggested that curcumin, curcumenone, curcumenol and zederone could be used as unique chemical markers for *C. longa*, *C. wenyujin*, *C. phaeocaulis* and C. *kwangsiensis*, respectively.

**Conclusions:**

This study developed a UHPLC/Q-TOFMS method coupled with multivariate statistical analysis to discriminate between *Curcumae* Radix samples from four different *Curcumae* species, i.e., *C. longa*, *C. wenyujin*, *C. phaeocaulis* and *C. kwangsiensis*. Notably, this new approach resulted in the identification of curcumin (**a**), curcumenone (**b**), curcumenol (**c**) and zederone (**d**) as unique chemical markers for the identification.

**Electronic supplementary material:**

The online version of this article (doi:10.1186/s13020-016-0095-8) contains supplementary material, which is available to authorized users.

## Background

Herbal medicines may be derived from specific single or multiple plant species, and a herb may have more than one species. The increasing popularity of multi-species herbs in medical use was documented in the 2010 edition of the Chinese Pharmacopoeia [1]. For instance, snow lotus herb (*Xuelian*), which has several health benefits, is mainly derived from *Saussurea involucrata*, *S. laniceps* and *S. medusa* [2], whereas dragon’s blood (*Xuejie*), which is used as a medicine, is mainly derived from *Daemonorops draco* and *Dracaena cochinchinensis* [3]. Previous studies have demonstrated that the chemical characteristics of multi-species herbal medicines can vary considerably depending on the originating species [3–5]. However, despite these findings, multi-species herbs are still treated as though they are identical. Each species of herb has its own genetic traits and geographical origins, which manifest themselves as unique primary and secondary metabolite patterns (i.e., metabolome) [3–5].

*Curcumae* Radix (*Yujin*) is used to treat a variety of different diseases, including hepatitis, cholecystitis, hyperlipidaemia and cancer [6–8]. Essential oils and curcuminoids are considered to be the major bioactive ingredients of *Curcumae* Radix [9], which is obtained from the dried tuberous roots of four *Curcuma* species, including *Curcumawenyujin*, *C. kwangsiensis*, *C. phaeocaulis* and *C. longa*, as officially described in the Chinese Pharmacopoeia [10]. Given that *Curcumae* Radix can be derived from four different *Curcuma* species, there could be considerable differences in the chemical composition among these four species, which differences could affect their therapeutic effects. It is difficult to distinguish between the origins of raw materials in the clinic because of their similar morphological features. To date, several analytical methods, including liquid chromatography-mass spectrometry (LC-MS) [11, 12], high-performance liquid chromatography (HPLC) [13, 14], gas chromatography-mass spectrometry (GC-MS) [15, 16], thin layer chromatography (TLC) [17] and capillary electrophoresis (CE) [18], have been developed to discriminate among the different species of *Curcuma* based on the chemical diversity of several main ingredients, particularly sesquiterpenoids. Metabolomics could be readily used to qualitatively and quantitatively monitor variations in the chemical component profiles of herbal medicines derived from different species [19, 20], as well as those subjected to different processing methods [21, 22], cultivation times [23] and geographical locations [24]. Recently, a GC-MS-based metabolomics method was successfully established to discriminate three *Curcuma* species according to the global chemical difference in *Curcuma* rhizomes (*Ezhu*) [19]. However, several sesquiterpene compounds from *Curcuma* plants are heat-sensitive. For example, furanodiene degrades to curzerene via a [3,3]-sigmatropic reaction (Cope rearrangement) upon heat treatment [25], whereas (4*S*, 5*S*)-germacrone-4,5-epoxide cyclizes through a transannular reaction on exposure to heat [26].

The aim of this study was to characterize the chemical profiles of *Curcumae* Radix and develop a rapid method to discriminate between the species by ultra-high-performance liquid chromatography–quadrupole time-of-flight mass spectrometry (UHPLC/Q-TOFMS) combined with multivariate statistical analysis.

## Methods

### Plant materials and chemical reagents

Thirty-three batches of authentic *Curcumae* Radix were collected from Good Agricultural Practice (GAP)-certified farms located in Rui’an and Leqing, Zhejiang province (C. *wenyujin*, n = 12), Yulin and Linshan, Guangxi province (*C. kwangsiensis*, n = 6), and Leshan and Chengdu, Sichuan province (*C. phaeocaulis*, n = 6; and *C. longa*, n = 9) provinces of China during December, 2012. The botanical origins of the different *Curcumae* Radix samples were morphologically recorded [27] based on the whole plants when they were collected from the GAP-certified farms, and were subsequently identified by Dr. Fengqing Yang at the School of Chemistry and Chemical Engineering, Chongqing University, China. Voucher specimens of the *Curcumae* Radix samples from four different *Curcuma* species (No. YJ01-YJ33) were deposited at the Institute of Chinese Medical Sciences, University of Macau, Macao, China. HPLC-grade methanol and acetonitrile were purchased from Baker Company (Sanford, ME, USA). LC-MS grade formic acid was purchased from Sigma-Aldrich (St. Louis, MO, USA). All of the other chemicals and solvents used in the current study were purchased as the analytical grades and used as received. Ultra-high-purity water was prepared using a Millipore SAS-67120 (Molsheim, Cedex, France).

### Pressurized liquid extractions (PLE)

Samples for analysis were prepared using a Dionex ASE 200 system (Dionex, Sunnyvale, CA, USA) according to a previously described procedure [28]. Briefly, *Curcumae* Radix was dried at 60 °C for 2 h, and then pulverized in a mill to give a homogeneous powder (60 mesh). A portion of the dried powder (approximately 0.5 g) was mixed with diatomaceous earth (0.5 g), and the resulting mixture was transferred to an 11-mL stainless steel extraction cell. The sample was subsequently extracted under the following conditions: solvent, methanol; pressure, 6.89 × 10^3^ kPa (1000 psi); temperature, 100 °C; static extraction time, 5 min; number of extractions, 1; and flush volume, 40 %. After the extraction, the PLE extract was transferred to a 25-mL volumetric flask, which was made up to its volume with methanol. An aliquot of the extract was centrifuged (Heraeus Multifuge; Thermo Fisher Scientific, USA) at 11,200×*g* for 5 min, and the supernatant was filtered through a 0.22-μm polytetrafluoroethylene filter (Whatman, NJ, USA) prior to being injected into the UHPLC/Q-TOFMS system.

### LC-MS analysis

UHPLC analysis was performed by an ACQUITY UHPLC system (Waters, Milford, MA, USA) equipped with a binary solvent delivery manager, auto-sampler and high temperature (HT) column oven. The UHPLC system was also equipped with an ACQUITY HSS T3 C_18_ column (100 × 2.1 mm i.d., 1.8 µm; waters) for the chromatographic separation of the sample mixture. The column was eluted with a binary gradient elution system consisting of (solvent A) acetonitrile and (solvent B) 0.1 % aqueous formic acid solution (containing 1 % acetonitrile). Chromatographic separation was achieved under the following elution conditions: isocratic 1 % solvent A (0–0.5 min); linear gradient from 1 % to 99 % solvent A (0.5–5 min); isocratic 99 % solvent A (5.0–6.0 min); and linear gradient from 99 % to 1 % solvent A (6.0–8.0 min). The flow rate and column temperature were set at 0.45 mL/min and 45 °C, respectively. An aliquot (5 µL) of each sample was injected into the column.

The UHPLC system was connected to a Xevo G2 quadrupole time-of-flight (Q-TOF) mass spectrometer (waters) with an electrospray ionization (ESI) source. Samples of the eluent from the UHPLC system were analyzed on this system under the following conditions: ionization mode, positive; source temperature, 120 °C; capillary voltage, 3000 V; sampling cone voltage, 30 V; extraction cone voltage, 4 V; cone gas flow, 20 L/h; desolvation gas flow, 800 L/h; and desolvation temperature, 450 °C. Mass data were collected in the range of *m/z* 50–1200 Da in the centroided mode. A lock-mass calibrant of leucine-enkephalin was continuously introduced to the mass spectrometer at a concentration of 200 ng/mL via a lock-spray interface at a flow-rate of 50 μL/min. This process generated a reference ion in the positive ionization mode (i.e., [M+H]^+^ = 556.2771), which ensures a high level of accuracy during the MS analysis.

### Method validation

A pooled sample of *Curcumae* Radix extracts was prepared by mixing 200 μL of each batch of *Curcumae* Radix extract in a single sample vial. The resulting mixture was used to provide a representative quality control (QC) sample containing all of the analytes at an average concentration. The QC sample was analyzed six times at the beginning of each run to ensure that the system was properly equilibrated. The QC sample was also analyzed after every three tested samples to further monitor the stability of the analytical process based on three representative ions in the chromatogram (i.e., *t*_R_ 2.50 min, *m/z* 312.1601; *t*_R_ 4.16 min, *m/z* 219.1744; and *t*_R_ 4.80 min, *m/z* 231.1385), which were selected for the validation of the method in the positive ionization mode. The relative standard deviations (RSDs) of peak intensity, retention time and molecular weight for the selected ions in the pooled QC samples were calculated (RSD = SD/mean)] to evaluate the reproducibility and stability of the method.

### Data processing and pattern recognition

The raw UHPLC/Q-TOFMS data were imported to the Progenesis QI software (Waters) for peak detection and alignment using the following parameters: mass tolerance within 5 ppm; retention time tolerance within 0.3 min; and relative mass error of theoretical fragmentation within 5 ppm. All data were normalized to the summed total ion intensity per chromatogram and three-dimensional data matrices were generated consisting of variable ID numbers (retention time-*m/z* value), sample codes and normalized peak areas. These matrices were then entered into the SIMCA-P 13.0 software (Umetrics AB, Sweden) for multivariate statistical analysis, including unsupervised principal component analysis (PCA). The resulting PCA model was evaluated and interpreted in terms of the *R*^*2*^*X* (cum) and *Q*^*2*^ (cum) values in its score plot. The *R*^*2*^*X* value represents the explanatory capacity of the variables, whereas *Q*^*2*^ provides an indication of the predictive capability of the model. The *R*^*2*^*X* and *Q*^*2*^ values were both found to be close to 1.0, indicating the good fitness of this method. Various chemical components were extracted from the loading plot of the PCA and a scatter plot (*S*-plot) of OPLS-DA as being responsible for the difference between the *Curcuma* species. Furthermore, ions with high variable importance on projection (VIP) values from the *S*-plots were considered to be potential chemical biomarkers, and subsequently subjected to further structural identification. A one-way ANOVA was also employed to test the significance of any differences observed between these markers among the four different species of *Curcuma* Radix. Differences with *P* ≤ 0.05 were considered statistically significant.

## Results and discussion

### Method development and validation

**Fig. 1:**
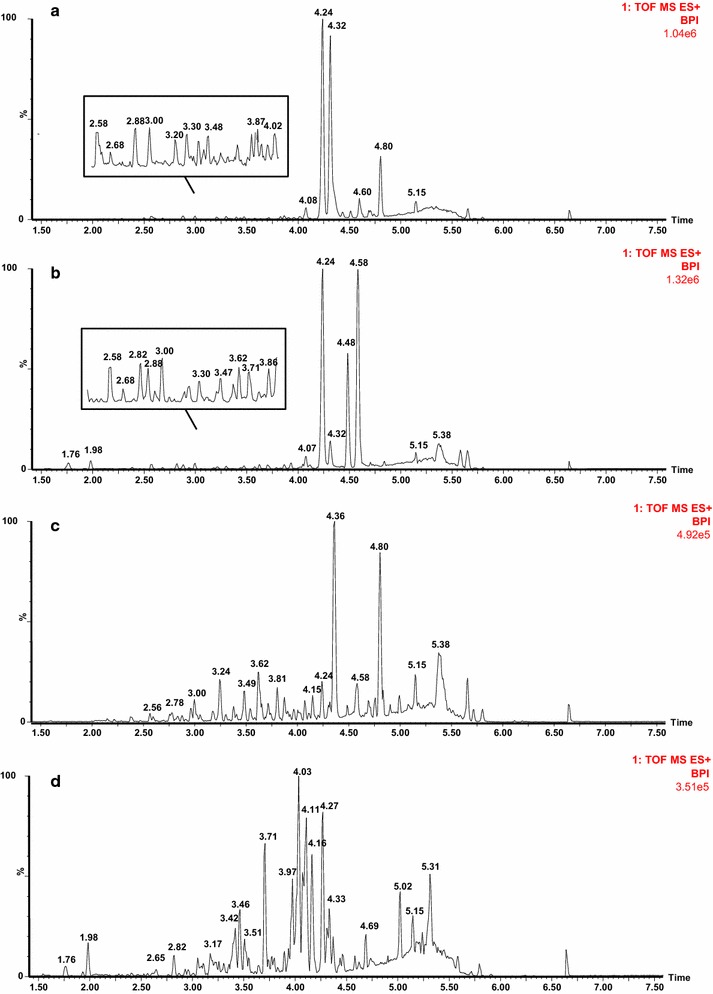
Base peak intensity (BPI) chromatograms of representative *Curcuma* Radix derived from (**a**) *C. phaeocaulis*, (**b**) *C. wenyujin*, (**c**) *C. kwangsiensis* and (**d**) *C. longa*, analyzed by UPLC/Q-TOFMS in positive ion mode

It can be difficult to distinguish between the different types of compound found in the different species of *Curcumae* Radix (e.g., the essential oils and curcuminoids) using the short analytic times associated with UHPLC. With this in mind, we conducted a small pilot study to optimize the chromatographic conditions used for the UHPLC analysis, including the column type and mobile phase, to achieve the highest possible resolution with the fastest separation time. Three ACQUITY UHPLC columns were evaluated in the current study, including BEH C18 (100 × 2.1 mm i.d., 1.7 μm), BEH HILIC (50 × 2.1 mm i.d., 1.7 μm) and HSS T3 C18 (100 × 2.1 mm i.d., 1.8 μm) columns. Among these columns, the ACQUITY HSS T3 C18 column was found to be the most suitable for the analysis of *Curcumae* Radix because it afforded good peak capacity and the best resolution of the major components. A variety of different mobile phases were evaluated for the elution of the column, including acetonitrile–water and methanol–water systems. These mobile phase systems were also evaluated in the presence of various modifiers, and the results were compared to determine which system gave the best resolution. The results of this screening process revealed that a combination of acetonitrile (organic mobile phase) and 0.1 % aqueous formic acid (containing 1 % acetonitrile; aqueous mobile phase) was optimal for the simultaneous separation of the major components in *Curcumae* Radix, as well as being compatible with the MS analysis (i.e., no ionization suppression from the formic acid). The addition of 1 % acetonitrile to the aqueous phase was found to be beneficial for stabilizing the baseline drift of instrument analysis. Ionization was conducted in the positive and negative ionizations modes to achieve maximum signal strength. It is noteworthy that higher levels of sensitivity and more structural information were obtained in the positive ionization mode. Having established the optimized chromatographic and MS conditions, we were able to successfully separate and identify the major chemical components in *Curcumae* Radix within 8 min (Fig. 1). To validate this method, we measured the RSD of the peak areas and *m/z* values (i.e., [M+H]^+^) of selected peaks (i.e., *t*_R_ 2.50 min, *m/z* 312.1601; *t*_R_ 4.16 min, *m/z* 219.1744; and *t*_R_ 4.80 min, *m/z* 231.1385), which were less than 5.46 and 0.01 %, respectively. Furthermore, the retention times of these peaks remained exactly the same over 16 runs, which indicated that the established method was robust with good reproducibility and stability for the analysis of experimental *Curcumae* Radix samples.

### Multivariate statistical analysis

Visual inspection of the results revealed that there were obvious differences among the four different *Curcumae* species. However, it was not possible to differentiate between these four groups based on visual inspection alone. We therefore applied multivariate statistical analysis to intuitively refine intergroup differences in the chemical components and identify potential chemical biomarkers that could be used to distinguish between the different groups.

**Fig. 2:**
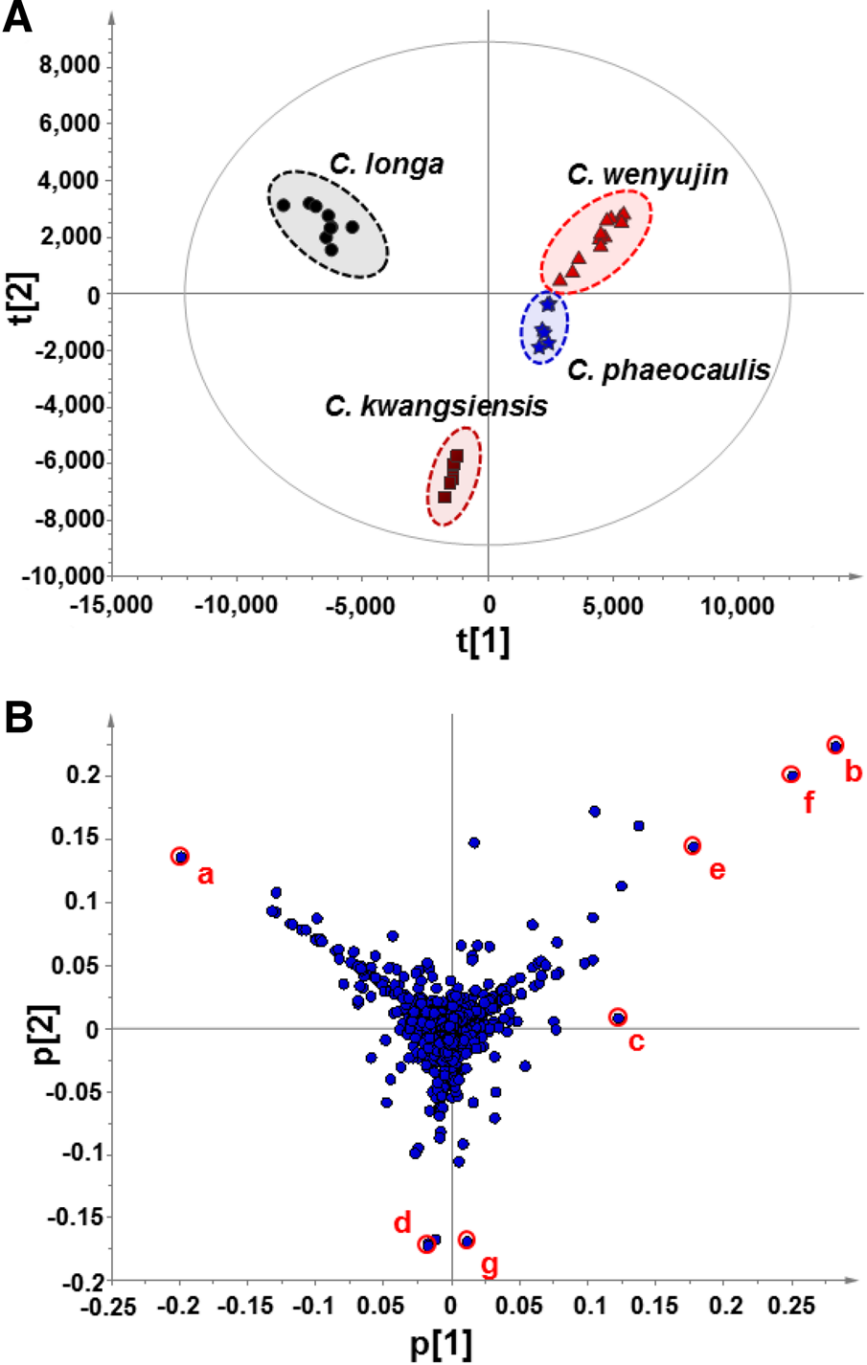
PCA/Scores plot (**A**) and loading plot (**B**) based on the global chemical profiling of *Curcuma* Radix derived from four species. The components contributing most to the differences were marked as a *red circle*, including (*a*) *t*_R_ 4.11 min, *m/z* 369.1331; (*b*) *t*_R_ 4.24 min, *m/z* 235.1696; (*c*) *t*_R_ 4.31 min, *m/z* 235.1697; (*d*) *t*_R_ 4.35 min, *m/z* 247.1333; (*e*) *t*_R_ 4.48 min, *m/z* 237.1851; (*f*) *t*_R_ 4.58 min, *m/z* 237.1853; and (*g*) *t*_R_ 4.80 min, *m/z* 231.1383

The resulting dataset contained sample code, *t*_R_–*m/z* pair and ion intensity data, which were processed by PCA to clearly discriminate between the four species based on the differences in their chemical compositions. It is noteworthy that a total of 2599 *t*_R_–*m/z* pairs were extracted in the positive ion mode for all of the samples analyzed in this study. After Pareto scaling and mean-centering, these data were displayed as scores and loadings in a coordinate system of principal components resulting from data dimensionality reduction. The three-component PCA score plots (Fig. 2A) showed that the 33 *Curcumae* Radix samples could be clearly classified into four different clusters depending on their species, according to the differences in their global chemical profiles. Although the clusters corresponding to *C. wenyujin* and *C. phaeocaulis* were close to each other, they could be clearly distinguished by PC2. Sevenfold cross validation was used to assess the validity of the model. Notably, all of the observations in the current study fell within the Hotelling T2 (0.95) ellipse. Furthermore, the *R*^2^*X* (cum) and *Q*^2^ (cum) values were determined to be 0.788 and 0.724, respectively, thereby highlighting the quality of our PCA model.

### Characterization of chemical markers

A loading plot of the PCA was constructed to identify the characteristic chemical components responsible for the differences observed across the four species of *Curcumae* Radix. Each point in the loading plot represents a chemical component (variable), and the further each variable moves away from the main cluster of the analyzed chemicals, the more likely it is that this component makes a significant contribution to the intergroup differences. As shown in Fig. 2B, the loading plot of the PCA revealed that seven ions, including **a** (*t*_R_ 4.11 min, *m/z* 369.1331), **b** (*t*_R_ 4.24 min, *m/z* 235.1696), **c** (*t*_R_ 4.31 min, *m/z* 235.1697), **d** (*t*_R_ 4.35 min, *m/z* 247.1333), **e** (*t*_R_ 4.48 min, *m/z* 237.1851), **f** (*t*_R_ 4.58 min, *m/z* 237.1853) and **g** (*t*_R_ 4.80 min, *m/z* 231.1383), were making significant contributions to the clusters. These ions were therefore considered to be potential chemical markers for discriminating between the four species of *Curcumae* Radix.

**Fig. 3:**
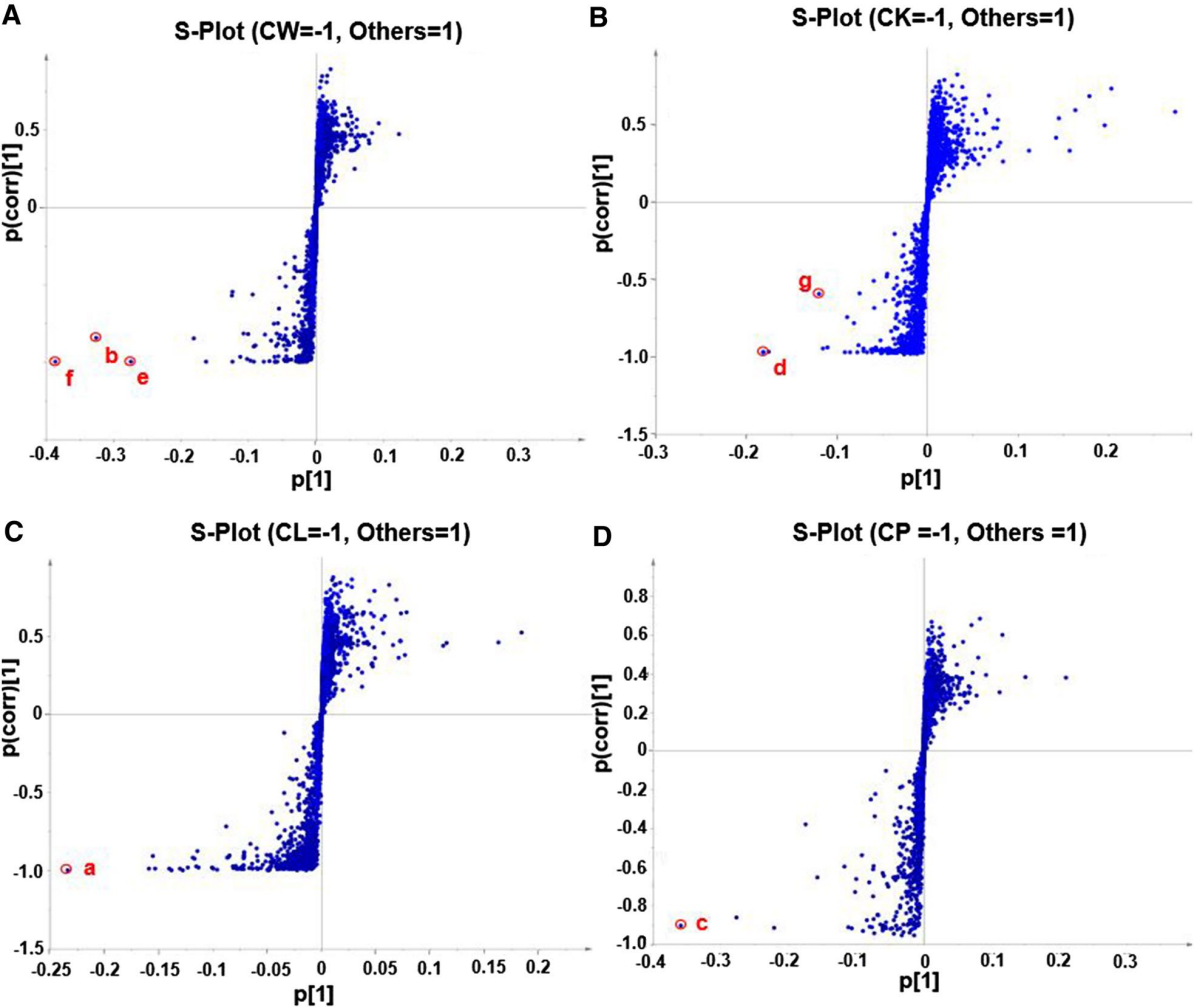
*S*-plots of OPLS-DA constructed based on a comparison of *C. phaeocaulis* (CP, **A**), *C. wenyujin* (CW, **B**), *C. kwangsiensis* (CK, **C**) and *C. longa* (CL, **D**) with the remaining three species. (**a**) *t*_R_ 4.11 min, *m/z* 369.1331; (**b**) *t*_R_ 4.24 min, *m/z* 235.1696; (**c**) *t*_R_ 4.31 min, *m/z* 235.1697; (**d**) *t*_R_ 4.35 min, *m/z* 247.1333; (**e**) *t*_R_ 4.48 min, *m/z* 237.1851; (**f**) *t*_R_ 4.58 min, *m/z* 237.1853; and (**g**) *t*_R_ 4.80 min, *m/z* 231.1383

An *S*-plot of the OPLS-DA was constructed based on a comparison of the results for one species with those of the remaining three species to confirm the identities of the chemical markers obtained from the loading plots, as well as identifying the characteristic chemicals of each species (Fig. 3). In this particular case, we used *C. wenyujin* as an example. The 12 *Curcumae* Radix samples derived from *C. wenyujin* were defined as groups, whilst the remaining 21 samples were marked as the second group, which formed the basis of our OPLS-DA model. The resulting *S*-plot is shown in Fig. 3A, where each point indicates an ion *t*_R_-*m/z* pair. The X and Y axes represent the contribution and confidence of the variables, respectively. In this plot, the further a specific data point is from zero on the X or the Y axis, the greater its contribution or confidence level for the two-group separation, respectively. The points at either ends of the S-shaped curve therefore represent potential chemical markers with the highest confidence [29]. In terms of the results, three ions (**b**, **e** and **f**) were found at the end of the S-shaped curve in the bottom-left corner of the graph, which were considered to be characteristic components that made the greatest contribution to distinguish *C. wenyujin* from the other three species. These components were responsible for the variance observed in the *Curcumae* Radix derived from *C. wenyujin* versus the other three species. Two ions (**d**, **g**) were found to be characteristic markers capable of discriminating *C. kwangsiensis* from the other species of *Curcumae* Radix (Fig. 3B). In contrast, ions **a** and **c** were found to be specific markers for the *Curcumae* Radix derived from *C. longa* and *C. kwangsiensis*, respectively.

### Identification of chemical markers and their changes in relative intensities

**Fig. 4:**
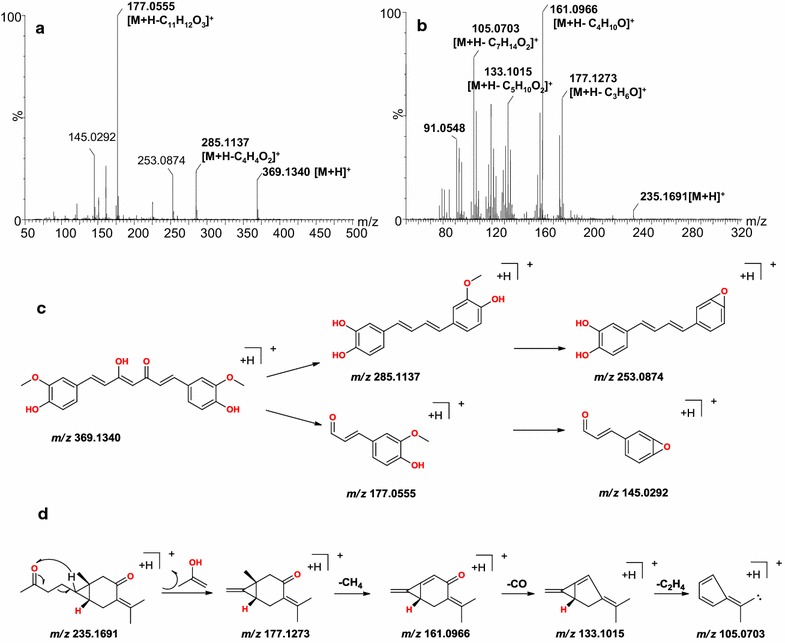
Mass spectra for the proposed fragmentation pathways of curcumin (**a**, **c**) and curcumenone (**b**, **d**) in the positive ionization mode

The Mass Fragment™ software package from waters was used to elucidate the structures of the different chemical components based on their fragmentation patterns. In this study, we were only able to identify seven chemical markers using the loading plots and the *S*-plots (Fig. 4). Furthermore, these structures were only tentatively assigned based on a comparison of their molecular weights, molecular formulas and MS/MS fragment ions with those published in the literature for compounds [11–16] isolated from *Curcuma* species, as well a comparison with known biochemical databases, such as MassBank (http://www.massbank.jp/), PubChem (http://pubchem.ncbi.nlm.nih.gov/), and Respect for Phytochemicals (http://spectra.psc.riken.jp/). The error in the measured molecular mass values was determined to be less than 2 mDa based on a comparison with the theoretical exact mass values provided by the authoritative websites mentioned above. Representative MS/MS spectra, chemical structures and proposed fragmentation pathway details for chemical marker **a** are shown in Fig. 4a. MS analysis of chemical marker **a** revealed an *m/z* value of 369.1340 for [M+H]^+^ in the positive ionization mode, which suggested that its empirical molecular formula was C_21_H_20_O_6_. The differences in the mass of this parent ion and its two main fragment ions (*m/z* 285.1137 and 177.0555) were 80 and 192 Da, which corresponded to loss of the C_4_H_4_O_2_ and C_11_H_12_O_3_, respectively. These data suggested that this compound was curcumin, based on a comparison with data from the literature [12, 30]. The most plausible interpretation of this fragmentation pathway is shown in Fig. 4c, which is primarily based on the information from the MS/MS data. Similarly, MS analysis of ion **b** (Fig. 4b) revealed an *m/z* value of 235.1699 for [M+H]^+^ in the positive ionization mode, which suggested that this compound had an empirical molecular formula of C_15_H_23_O_2_. Ion **b** gave a fragment ion with an *m/z* value of 177.1273, representing a 58 Da mass difference compared with the parent ion, indicating the loss of C_3_H_6_O. This daughter ion (*m/z* 177.1273) afforded four main fragment ions with *m/z* values of 161.0966, 133.1015, 105.0861 and 91.0548, representing mass differences of 16, 44, 58 and 62 Da, respectively, which corresponded to the successive loss of the CH_4_, CO, CH_2_ and CH_2_. These data therefore suggested that this compound was curcumenone and its fragmentation pathway is shown in Fig. 4d [31]. Based on the method described above, markers **c**–**g** were tentatively identified as curcumenol, zederone, neocurdione, curdione and curzerenone, respectively, by comparing their data with those from the literature [31, 32], as well as searching the biochemical databases listed above (Additional file 1).

**Fig. 5:**
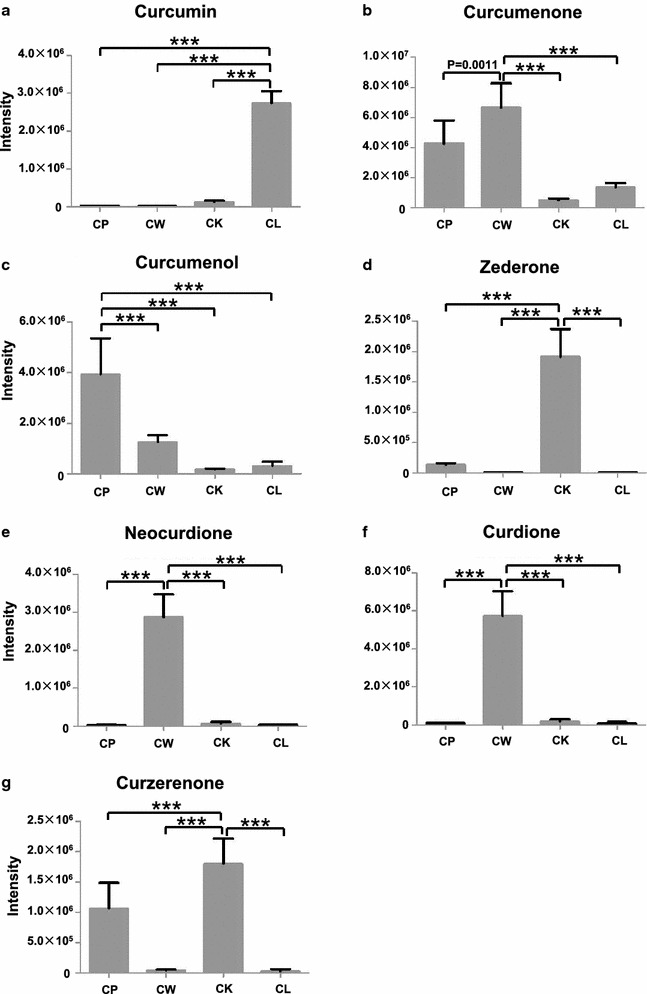
Relative intensities of the chemical markers in *Curcuma* Radix derived from four species. (**a**) curcumin, (**b**) curcumenone, (**c**) curcumenol, (**d**) zederone, (**e**) neocurdione, (**f**) curdione and (**g**) curzerenone. Data represent the mean values ± SD (n = 3). ** and **** indicate *P* < 0.01 and *P* < 0.0001, respectively

The signal intensities of the chemical markers identified in the different *Curcumae* Radix samples were analyzed by one-way ANOVA to provide a further comparison of the differences among the four species of *Curcumae* Radix. The marker levels varied considerably among the different *Curcumae* Radix samples (Fig. 5). The intensity of curcumin was found to be significantly (*P* < 0.0001) higher in *C. longa* than it was in any of the other three species (Fig. 5a), suggesting that curcumin could be used as a specific marker for distinguishing *C. longa* from the other three species. *C. wenyujin* showed significantly higher levels (*P* < 0.001) of curcumenone (**b**), neocurdione (**e**) and curdione (**f**) than the other three species of *Curcumae* Radix (Fig. 5b, e and f). Furthermore, *C. wenyujin* contained higher levels of neocurdione and curdione (35- to 140-times higher) than any of the other three species of *Curcumae* Radix, suggesting that these compounds could be considered as markers for distinguishing *C. wenyujin* from the other three species. Curcuminoids and sesquiterpenoids are considered to be the main bioactive ingredients of *Curcumae* Radix [13, 14]. Curcuminoids, such as curcumin, have been reported to exhibit a wide range of interesting pharmacological activities, including antioxidative, anti-inflammatory, hypocholesterolemic, antihepatotoxic and anticancer effects [4]. Sesquiterpenoids, such as curcumenone, curdione and curzerenone, are the main components of the essential oils derived from *Curcumae* Radix, which have been reported to exhibit antioxidative, anti-tumor and antiviral activities [32].

### PCA analysis based on the identified chemical markers

**Fig. 6:**
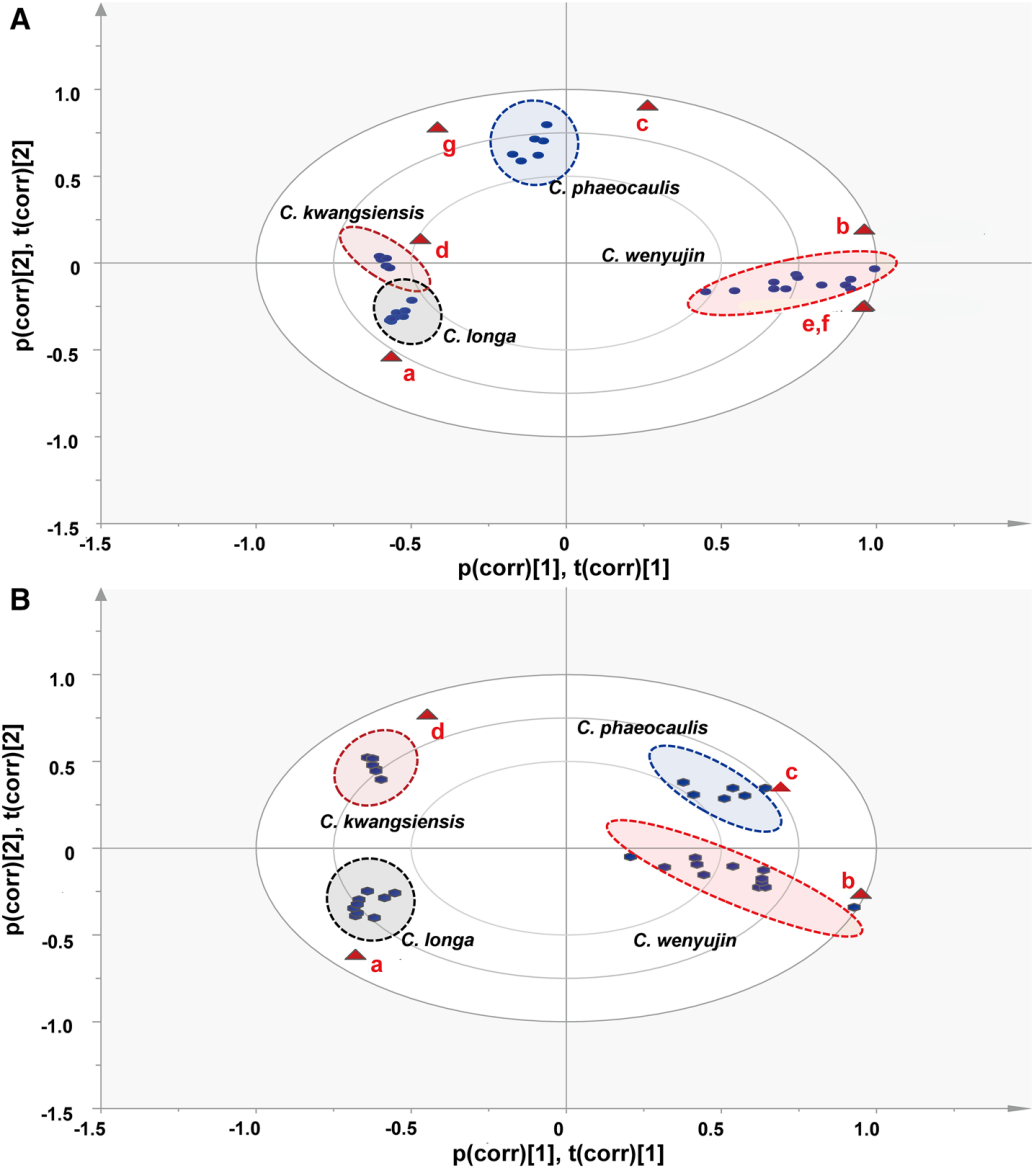
PCA/bi-plots of the 33 *Curcuma* Radix samples constructed using (**A**) seven chemical markers (**a**–**g**) and (**B**) four unique chemical markers (**a**–**d**). (**a**) curcumin, (**b**) curcumenone, (**c**) curcumenol, (**d**) zederone, (**e**) neocurdione, (**f**) curdione and (**g**) curzerenone

PCA was performed based on the seven chemical markers identified above to assess their ability to actively discriminate between the different species of *Curcumae* Radix. After normalizing the fused data, a 33 (objects) × 7 (variables) dataset was constructed and subjected to PCA. The resulting PCA bi-plot is shown in Fig. 6a, where the observations and variables of the multivariate data are represented in the same plot. These data showed that variables with similar loadings appeared to be strongly correlated with the objects. In a similar manner to Fig. 2A, all of the *Curcumae* Radix samples were successfully separated into their correct species based on the differences in these seven chemical markers.

Based on the VIP values obtained from the *S*-plots of the OPLS-DA models (Fig. 3), curcumin (**a**), curcumenone (**b**), curcumenol (**c**) and zederone (**d**) were selected as unique markers for *C. longa*, *C. wenyujin*, *C. phaeocaulis* and *C. kwangsiensis*, respectively. PCA was also conducted based on these four unique markers using a 33 (objects) × 4 (variables) data matrix, and the resulting PCA bi-plot revealed that all of the *Curcumae* Radix samples evaluated in the current study were successfully separated into the correct species based on these four markers. It is noteworthy that the separation observed between *C. kwangsiensis* and *C. longa* (Fig. 6B) was much clearer for the four chemical markers than it was for the PCA bi-plot constructed using the seven chemical markers described above (Fig. 6A). Our data therefore suggest that curcumin (**a**), curcumenone (**b**), curcumenol (**c**) and zederone (**d**) are unique chemical markers for *C. longa*, *C. wenyujin*, *C. phaeocaulis* and *C. kwangsiensis*, respectively, which could be used to discriminate between different *Curcumae* Radix samples in terms of their species of origin.

## Conclusions

This study developed a UHPLC/Q-TOFMS method coupled with multivariate statistical analysis to discriminate between *Curcumae* Radix samples from four different *Curcumae* species, i.e., *C. longa*, *C. wenyujin*, *C. phaeocaulis* and *C. kwangsiensis*. This method was used to identify curcumin (**a**), curcumenone (**b**), curcumenol (**c**) and zederone (**d**) as unique chemical markers of four *Yujin*.

## Additional file

10.1186/s13020-016-0095-8 Mass spectra in the positive ionization mode and chemical structures of (**A**) curcumenol and (**B**) zederone, (**C**) neocurdione, (**D**) curdione and (**E**) curzerenone.

## Abbreviations

BPI: based peak intensity
CE: capillary electrophoresis
GAP: good agricultural practice
GC-MS: gas chromatography-mass spectrometry
HPLC: high-performance liquid chromatography
TLC: thin layer chromatography
*m/z*: mass-to-charge ratio
OPLS-DA: orthogonal partial least squared discriminant analysis
PCA: principal component analysis
PLE: pressurized liquid extractions
QC: quality control
RSDs: relative standard deviations
RT or t_R_: retention time
CMs: chinese medicines
UPLC/Q-TOFMS: ultra-performance liquid chromatography–quadrupole time-of-flight mass spectrometry
VIP: variable importance on projection
