# Supplementary figures and images for: Discriminating from species of Curcumae Radix (Yujin) by a UHPLC/Q-TOFMS-based metabolomics approach
Source: Chin Med. 2016 Apr 29;11:21. doi: 10.1186/s13020-016-0095-8 (PMC4850745; doi:10.1186/s13020-016-0095-8)

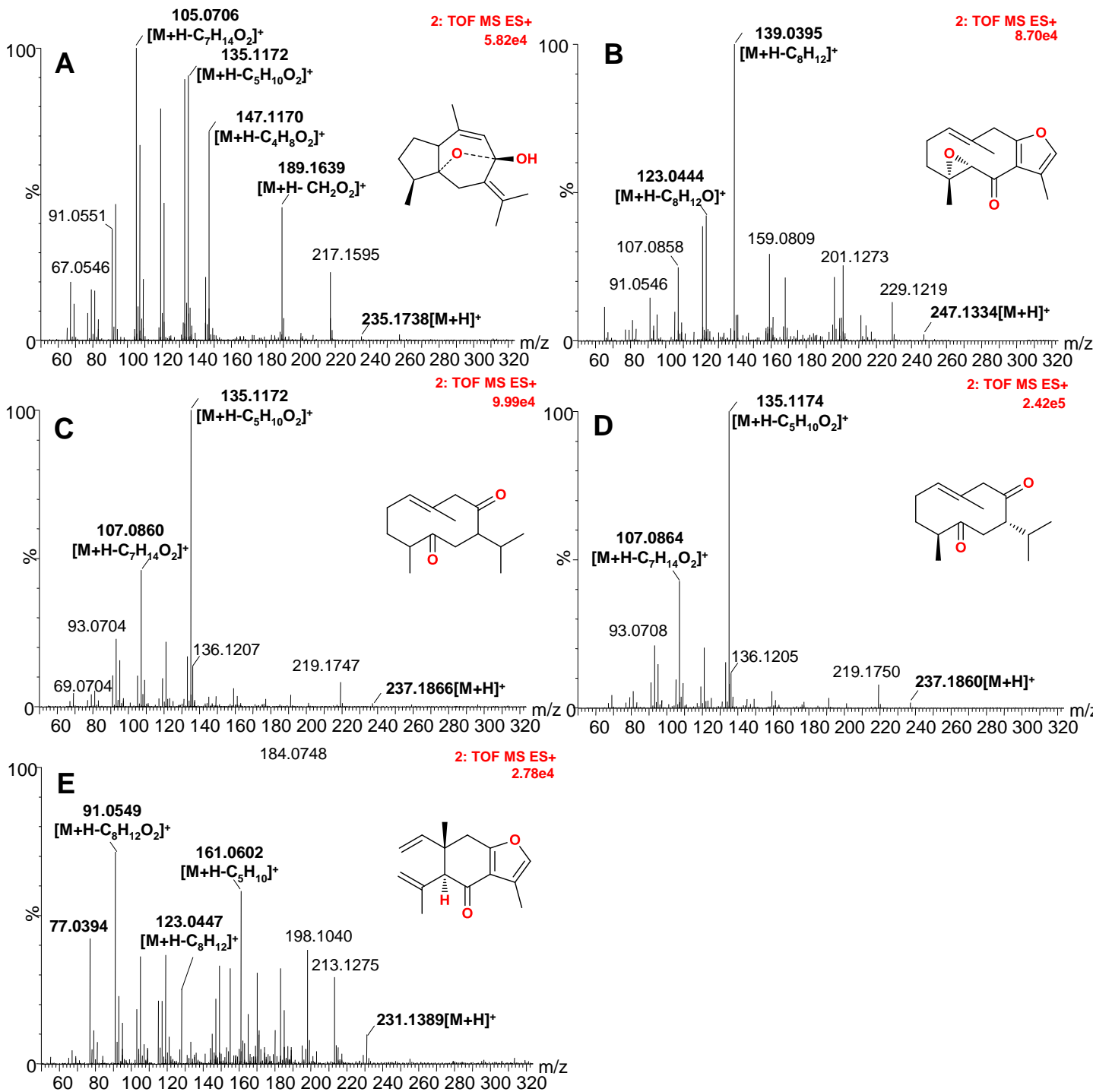

Supplement: Supplementary file 1 — 10.1186/s13020-016-0095-8 Mass spectra in the positive ionization mode and chemical structures of (A) curcumenol and (B) zederone, (C) neocurdione, (D) curdione and (E) curzerenone. [file 13020_2016_95_MOESM1_ESM.pdf]
